# Validation of a new *in vitro* Sun Protection Factor method to include a wide range of sunscreen product emulsion types

**DOI:** 10.1111/ics.12625

**Published:** 2020-07-27

**Authors:** M. Pissavini, C. Tricaud, G. Wiener, A. Lauer, M. Contier, L. Kolbe, C. Trullás Cabanas, F. Boyer, E. Meredith, J de Lapuente, E. Dietrich, P.J. Matts

**Affiliations:** ^1^ Coty‐Lancaster SAM 2, rue de la Lujerneta Monaco 98000 Monaco; ^2^ L’Oréal 188 rue Paul Hochart Chevilly Larue 94550 France; ^3^ Edgewell Personal Care Ormond Beach FL 32174 USA; ^4^ Chanel PB 135 avenue Charles de Gaulle Neuilly sur Seine 92200 France; ^5^ LVMH Recherche Parfums et Cosmétiques 185 avenue de Verdun Saint Jean de Braye 45800 France; ^6^ Beiersdorf AG Unnastrasse 48 Hamburg 20245 Germany; ^7^ ISDIN Provençals 33 Barcelona Spain; ^8^ Pierre Fabre Dermo Cosmetique Hôtel Dieu 2 Rue Viguerie BP3071 Toulouse cedex 3 31 025 France; ^9^ CTPA Sackville House 40 Piccadilly London W1J 0DR UK; ^10^ AC MARCA Avenida Carrilet 293‐297 L’Hospitalet del Llobregat Barcelona 08907 Spain; ^11^ Cosmetics Europe ‐ The Personal Care Association Avenue Herrmann‐Debroux 40 Brussels B‐1160 Belgium; ^12^ Procter & Gamble Greater London Innovation Centre Rusham Park, Whitehall Ln Egham TW20 9NW UK

**Keywords:** Sun Protection Factor, *in vitro*, ISO, ring test

## Abstract

In 2017, Cosmetics Europe performed a double‐blinded ring test of 24 emulsion‐type sunscreen products, across 3 *in vivo* test laboratories and 3 *in vitro* test laboratories, using a new candidate *in vitro* SPF test method. Based on the results of this work, an article was published showing how data derived from a new lead candidate method conform to new International Standards (ISO) acceptance criteria for alternative SPF test methods (Any alternative method should consider the matrix effect and if required, specify the matrix applicability of the method; Criterion 1a: Systematic differences between methods should be negligible: 95% of all individual results of an alternative method are within the range of ±2× reproducibility standard deviation of the *in vivo* method, that is overall bias must be below 0.5× reproducibility standard deviation of the *in vivo* method; Criterion 1b: Measurement uncertainty of an alternative method should be below the measurement uncertainty of the *in vivo* method. Candidate method predicted values must fall within the full ‘funnel’ (SPF 6‐50+) limits proposed by Cosmetics Europe (derived from the same minimum test design, that is using the ISO24444 Method to measure at least 24 products across at least 3 laboratories using at least 5 test subjects/laboratory, in a blinded fashion).). Of the 24 sunscreen products tested, the majority of emulsions were of the oil‐in‐water (O/W) type, whereas only one was water‐in‐oil (W/O) and there were no products with a mineral‐only sun filter system. In order to confirm the scope of this method, therefore, a new study was conducted that included 73 W/O (12 mineral + organic, 44 mineral only and 17 organic only) and 3 O/W mineral‐only, emulsion‐type sunscreen products (a total of 76 new sunscreen products). When combined with the previous 24 products (tested in 3 different laboratories), this yielded a new data set comprising a total of 100 emulsion‐type sunscreen products, with SPF values ranging from 6 to 50+ (with a total of 148 data points). These products were tested using the double‐plate *in vitro* SPF test method and compared with the ISO TC217/WG7 acceptance criteria for alternative SPF test methods. Over 95% of paired *in vitro*: *in vivo* SPF values lay within the upper and lower limits of the ISO acceptance criteria funnel, with no bias. This new *in vitro* SPF test method, therefore, meets the minimum requirements for an alternative SPF test method to ISO24444:2010, for emulsion‐type sunscreen products (which make up the majority of marketed sunscreen products).

## Graphical abstract

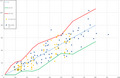

## Résumé

En 2017, Cosmetics Europe a réalisé un ring test en double aveugle de 24 produits de protection solaire de type émulsion, dans 3 laboratoires de test in vivo et 3 laboratoires de test in vitro, en utilisant une nouvelle méthode de test SPF in vitro. Sur la base des résultats de ces travaux, un article a été publié montrant comment les données dérivées de cette nouvelle méthode sont conformes aux nouveaux critères d'acceptation des normes internationales (ISO) pour les méthodes de test SPF alternatives. Sur les 24 produits de protection solaire testés, la majorité des émulsions étaient du type huile dans l'eau (H / E), tandis qu'un seul était de l'eau dans l'huile (E / H) et il n'y avait aucun produit contenant uniquement des minéraux. Afin de confirmer cette méthode, une nouvelle étude a donc été menée comprenant 73 produits E/ H (12 produits contenant des filtres minéraux + organiques, 44 produits contenant des filtres minéraux uniquement et 17 produits contenant des filtres organiques uniquement) et 3 produits H / E contenant des filtres minéraux uniquement, tous de type émulsion (donc un un total de 76 nouveaux produits de protection solaire). Combiné aux 24 produits précédents (testés dans 3 laboratoires différents), cela a donné un nouvel ensemble de données comprenant un total de 100 produits de protection solaire de type émulsion, avec des valeurs SPF allant de 6 à 50+ (avec un total de 148 points de données) . Ces produits ont été testés à l'aide de la méthode de test SPF in vitro double approche et comparés aux critères d'acceptation de l'ISO TC217 / WG7 pour les méthodes alternatives du SPF in vivo. Plus de 95% des valeurs de SPF appariées in vitro: in vivo se situent dans les limites supérieure et inférieure de l'entonnoir des critères d'acceptation ISO, sans biais. Cette nouvelle méthode de test SPF in vitro, par conséquent, répond aux exigences minimales d'une méthode de test SPF alternative à ISO24444: 2010, pour les produits de protection solaire de type émulsion (qui constituent la majorité des produits de protection solaire commercialisés).

## Introduction

In 2017, Cosmetics Europe performed a double‐blinded ring test of 24 emulsion‐type sunscreen products, across 3 *in vivo* test laboratories and 3 *in vitro* test laboratories, consistent with the Joint Research Center (the European Commission’s in‐house science service [1]) guidelines, ‘Selecting and/or validating analytical methods for cosmetics’, using a new candidate *in vitro* SPF test method. Based on the results of this work, an article was published showing how data derived from a new lead candidate method conform to the new ISO acceptance criteria (Table 1).

Of the 24 sunscreen products tested, the majority of emulsions were of the oil‐in‐water type (O/W), whereas only one was water‐in‐oil (W/O) and there were no products with a mineral‐only sun filter system (comprising varying ratios of micronized zinc and titanium dioxide).

In order to confirm the scope of this method, therefore, a new study was conducted that included 73 W/O (12 mineral + organic, 44 mineral only and 17 organic only) and 3 O/W mineral‐only, emulsion‐type sunscreen products (a total of 76 new sunscreen products), which were tested according to the protocol published previously [2].

## Materials and methods

### Sunscreen products

In addition to the 24 commercial primary, emulsion‐type, sunscreen products already used for the initial validation, 73 W/O (12 mineral + organic, 44 mineral only and 17 organic only) and 3 O/W mineral‐only emulsion sunscreens were chosen to represent the entire range of SPF categories defined by European Commission Recommendation 2006/647/EC [3] (namely 6, 10, 15, 20, 25, 30, 50 and 50+; see Table 1 for details).

**Table 1:** The 100 emulsion‐type sunscreen products used (SPF6 ‐ 50+)

Tested products	Emulsion type	Filters	Mean *in vivo* result	Individual *in vitro* result
				15.8
CE validation ring test P1	O/W	Organic	13.0	9.0
				11.6
				13.8
CE validation ring test P2	O/W	Organic	14.6	8.1
				10.0
				13.4
CE validation ring test P3	O/W	Organic	9.4	9.0
				9.7
				25.1
CE validation ring test P4	O/W	Organic	20.8	18.7
				14.3
				16.2
CE validation ring test P5	O/W	Organic	12.3	7.8
				10.1
				23.5
CE validation ring test P6	O/W	Organic	25.7	16.3
				27.4
				29.8
CE validation ring test P7	O/W	Mineral + organic	19.7	19.9
				19.8
				19.3
CE validation ring test P8	O/W	Organic	15.1	13.6
				16.9
				29.5
CE validation ring test P9	O/W	Organic	24.1	18.7
				23.3
				14.3
CE validation ring test P10	O/W	Mineral + organic	15.0	11.1
				13.0
				37.2
CE validation ring test P11	O/W	Mineral + organic	53.0	29.1
				36.0
				53.1
CE validation ring test P12	W/O	Organic	54.4	41.8
				65.3
				22.6
CE validation ring test P13	O/W	Mineral + organic	44.5	49.1
				44.2
				44.9
CE validation ring test P14	O/W	Mineral + organic	47.2	42.6
				64.2
CE validation ring test P15	O/W	Mineral + organic	57.1	62.3
				35.2
				53.4
				60.7
CE validation ring test P16	O/W	Mineral + organic	46.0	49.6
				61.3
				54.4
CE validation ring test P17	O/W	Organic	53.0	52.8
				71.0
				35.1
CE validation ring test P18	O/W	Mineral + organic	45.5	33.5
				41.6
				30.2
CE validation ring test P19	O/W	Mineral + organic	41.2	28.6
				56.3
				13.5
CE validation ring test P20	O/W	Organic	24.2	9.9
				12.4
				48.4
CE validation ring test P21	O/W	Organic	34.8	39.1
				41.3
				65.7
CE validation ring test P22	O/W	Mineral + organic	57.9	58.1
				58.6
				9.7
CE validation ring test P23	O/W	Organic	13.2	9.3
				14.3
				9.9
CE validation ring test P24	O/W	Organic	11.6	8.7
				9.6
P25	W/O	Organic	17.7	9.6
P26	W/O	Mineral + organic	55.4	39.0
P27	W/O	Organic	21.5	20.4
P28	W/O	Mineral only	12.5	8.9
P29	W/O	Mineral only	91.1	65.1
P30	W/O	Mineral only	33.1	40.4
P31	W/O	Mineral only	35.2	33.2
P32	W/O	Mineral + organic	54.7	30.5
P33	W/O	Mineral + organic	55.4	54.8
P34	W/O	Mineral + organic	41.7	34.8
P35	W/O	Mineral + organic	28.4	21.1
P36	W/O	Mineral + organic	28.8	18.7
P37	W/O	Mineral + organic	32.7	20.3
P38	W/O	Mineral + organic	57.3	57.1
P39	W/O	Mineral only	78.3	58.7
P40	W/O	Mineral + organic	17.9	14.8
P41	W/O	Mineral + organic	17.0	18.9
P42	W/O	Mineral only	9.0	13.3
P43	W/O	Mineral only	9.2	13.6
P44	W/O	Mineral only	9.8	9.2
P45	W/O	Mineral only	11.1	19.2
P46	W/O	Mineral only	12.1	21.1
P47	W/O	Mineral only	15.0	21.7
P48	W/O	Mineral only	15.3	7.7
P49	W/O	Mineral only	15.4	18.8
P50	W/O	Mineral only	18.2	20.4
P51	W/O	Mineral only	23.2	22.8
P52	W/O	Mineral only	23.5	35.2
P53	W/O	Mineral only	24.0	27.7
P54	W/O	Mineral only	25.7	29.3
P55	O/W	Mineral only	26.4	37.1
P56	W/O	Mineral only	26.4	24.8
P57	O/W	Mineral only	26.9	32.0
P58	W/O	Mineral only	29.2	31.6
P59	W/O	Mineral only	32.0	32.6
P60	W/O	Mineral only	32.9	25.2
P61	W/O	Mineral only	36.3	47.1
P62	W/O	Mineral only	36.6	27.4
P63	W/O	Mineral only	36.9	37.4
P64	W/O	Mineral only	37.6	40.8
P65	W/O	Mineral only	37.8	47.7
P66	O/W	Mineral only	38.8	48.6
P67	W/O	Mineral only	38.9	23.3
P68	W/O	Mineral only	41.2	72.5
P69	W/O	Mineral only	47.4	49.1
P70	W/O	Mineral only	47.8	43.4
P71	W/O	Mineral only	48.3	56.3
P72	W/O	Mineral only	50.0	63.6
P73	W/O	Mineral only	52.6	32.0
P74	W/O	Mineral only	54.3	51.3
P75	W/O	Mineral only	54.5	58.5
P76	W/O	Mineral only	57.3	58.3
P77	W/O	Mineral only	58.5	66.0
P78	W/O	Mineral only	59.1	48.6
P79	W/O	Mineral only	64.6	71.6
P80	W/O	Mineral only	69.3	62.4
P81	W/O	Mineral only	38.1	54.2
P82	W/O	Organic	75.3	80.3
P83	W/O	Mineral + organic	59.1	38.2
P84	W/O	Mineral only	40.2	28.3
P85	W/O	Organic	61.4	60.1
P86	W/O	Mineral only	39.4	33.6
P87	W/O	Organic	67.5	45.3
P88	W/O	Organic	51.4	66.3
P89	W/O	Organic	73.6	70.6
P90	W/O	Organic	65.0	58.7
P91	W/O	Organic	73.6	90.0
P92	W/O	Organic	67.5	65.0
P93	W/O	Organic	46.8	33.0
P94	W/O	Organic	70.3	70.6
P95	W/O	Organic	56.8	66.8
P96	W/O	Organic	74.1	103.4
P97	W/O	Organic	60.5	70.6
P98	W/O	Organic	80.7	66.8
P99	W/O	Organic	65.3	75.5
P100	W/O	Mineral + organic	36.7	51.3

It should be noted that *in vivo* data from the 76 new sunscreen products were not used to adjust the ISO acceptance criteria ‘funnel’.

### *In vivo* SPF test method

The 76 new sunscreen samples were tested on a minimum of 5 subjects using the current ISO24444:2010 *In Vivo* SPF test protocol [4], using a variety of test laboratories (according to the parent Company’s choice; the laboratories were previously audited by the Company supplying the test products).

### *In vitro* SPF test method

The method used in this study was exactly the same as described previously [5, 6]. The protocol is summarized below:

1. Preparation of reagents and materials
2. Product application on substrates and robot automatic spreading
3. Measurement of initial absorbance using two plate types (290 nm to 400 nm).
4. Calculation of initial *in vitro* SPF.
5. Calculation of irradiation dose (based on initial *in vitro* SPF).
6. Irradiation with calculated dose.
7. Measurement of final post‐irradiation absorbance using two plate types (290 nm to 400 nm).
8. Calculation of final *in vitro* SPF.

## Results and discussion

**Figure 1:**
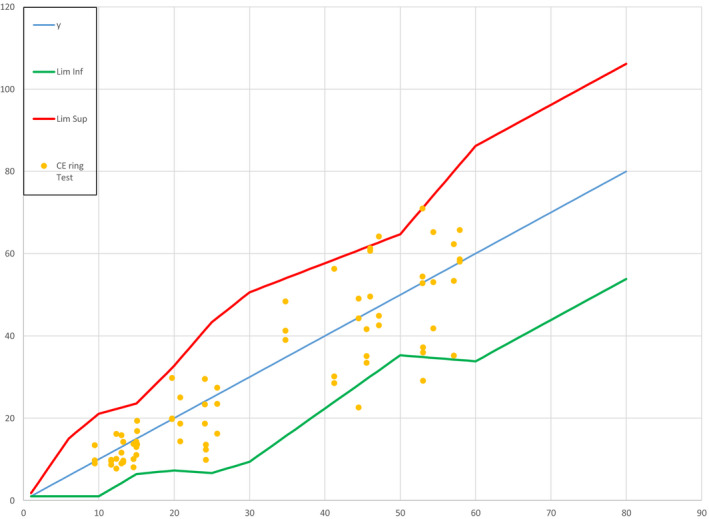
Results from blinded ring study, showing 72 data points laid over the ISO acceptance criteria ‘funnel’.

In the previous article, where we described the results of *in vitro* and *in vivo* testing of 24 products in 3 separate test laboratories [2], we showed that only 3 data points from the *in vitro*/*in vivo* relationship (out of a total of 72; 4.2%) lay outside the ISO acceptance criteria funnel, with no significant bias (see Fig. 1). The 95% confidence intervals of the slope of the *in vitro*/ *in vivo* relationship (0.85–1.17) included the expected value (that is, a perfect slope = 1.0), with a non‐significant intercept (−1.48; *P* = 0.62). Although these data met the requirements for the ISO Acceptance Criteria (95.8% of data points within the upper and lower limits of the funnel), this data set did not account for the full range of emulsion‐type sunscreen products in the marketplace (as they included a majority of O/W products and only one W/O product).

**Figure 2:**
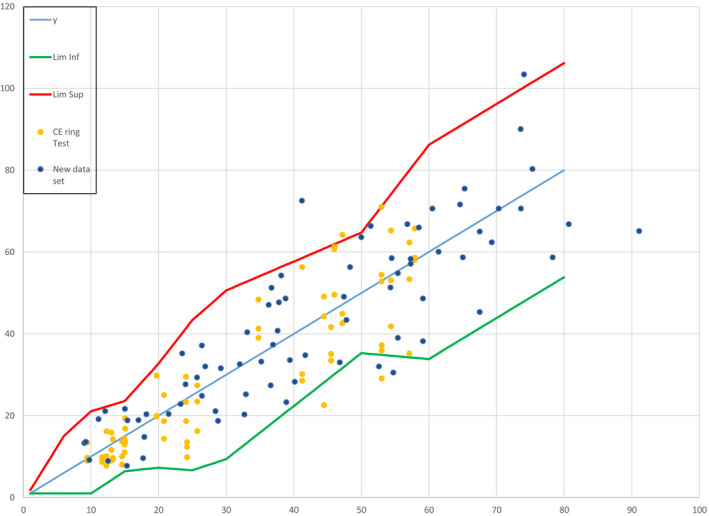
Results from ring study, showing 148 data points laid over the ISO acceptance criteria ‘funnel’.

When the data from testing the 73 W/O (12 mineral + organic, 44 mineral only, 17 organic only) and 3 O/W mineral‐only products were added to this plot (see Fig. 2), 7 data points (out of a new total of 148; 4.7%) lay outside the upper/lower limits of the acceptance criteria funnel.

**Figure 3:**
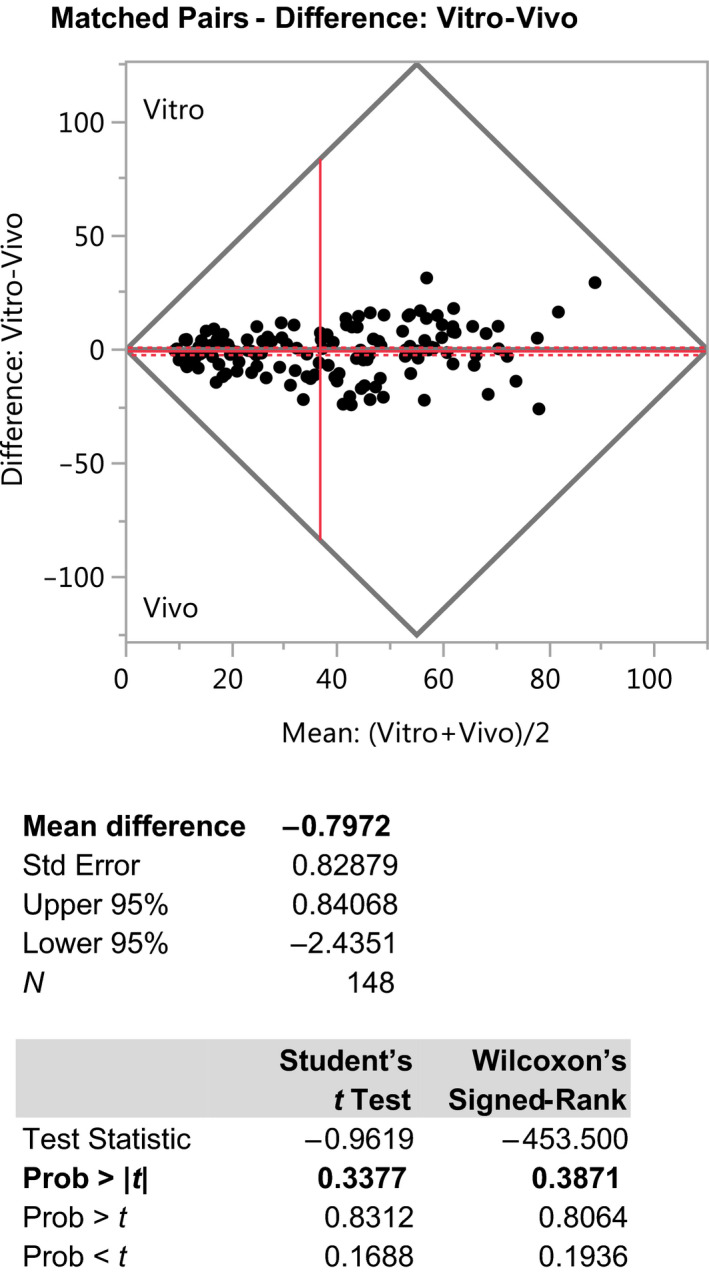
results of matched‐pairs analysis performed on the 148 couples of data (*in vivo* vs. *in vitro*).

A matched‐pairs analysis was performed on the 148 pairs of data (*In Vivo* vs. *In Vitro*) which showed no significant bias (Fig. 3; difference average equal to − 0.80, 95% CI −2.44 to 0.84, Student’s test *P*‐value = 0.34 and Wilcoxon’s test *P*‐value = 0.39).

In summary, therefore, the combined data sets from the previous study and this new study show that, across a full range of sunscreen product emulsion types (O/W and W/O; 100 products in total, covering the full range of SPF values marketed within Europe (6–50+), the *In Vitro* SPF Test Method still meets ISO Acceptance Criteria for alternative SPF test methods to the *in vivo* reference ISO24444:2010 SPF method.

**Figure 4:**
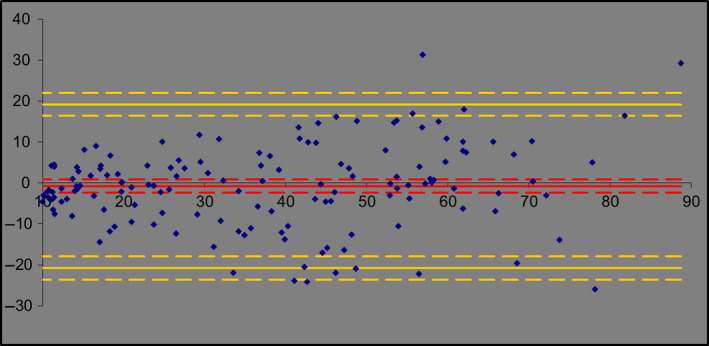
Bland–Altman plot of the 148 pairs of data (*in vivo* vs. *in vitro*). The red line shows the bias of the measures, the yellow lines show the 95% Limits of Agreement (LoA), and the dotted lines show the upper and lower limits of bias and 95% LoA.

Although we strongly believe that the ISO Acceptance Criteria funnel represents a robust model for testing the validity of alternative SPF test methods, we acknowledge that there are some who may prefer other means of demonstrating equivalence, such as a ‘Bland‐Altman’ plot [7] (a difference plot sometimes used in the fields of analytical chemistry or biomedicine to analyse the degree of agreement between two different assays). The resulting graph is an XY scatter plot, where the *y*‐axis represents the difference between two paired measurements (A‐B) and the *x*‐axis represents the average of these measures ((A + B)/2). In other words, the difference of the two paired measurements is plotted against the mean of the two measurements. Bland and Altman [8] recommended that 95% of the data points should lie within ± 2 SD of the mean difference. For completeness, therefore, we used the new data set to construct a Bland–Altman plot (see Fig. 4).

As 96.6% of the data points are contained with the upper/lower limits of the plot, the new *In Vitro* SPF test method meets of the success criteria for this method also.

**Figure 5:**
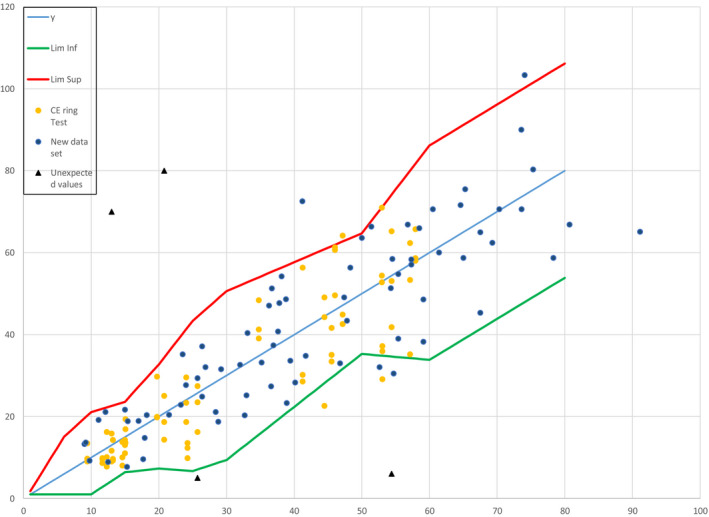
Funnel with 148 data points plotted, including measured data and 4 unexpected high or low values (shown as black triangles).

Discussing these results further, it is interesting to observe that, when unrealistically high/low data points are added to the data set (Fig. 5), the ‘funnel’ model rejects an hypothesis of agreement between the two methods (as 11 data points from the *in vitro*/*in vivo* relationship, 7.4% of a total of 148, lay outside the upper/lower limits).

**Figure 6:**
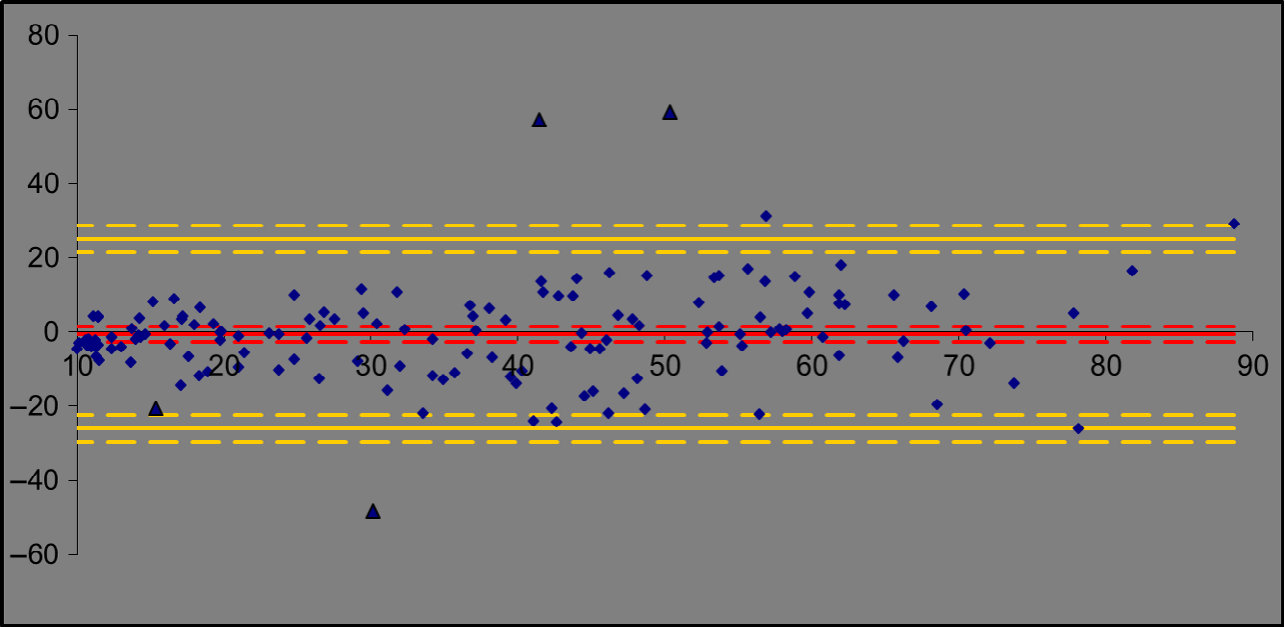
Bland–Altman method with 148 data points plotted, including measured data and 4 unexpected high or low values (shown as black triangles).

In contrast, when these values are added to the data set and analysed using the Bland–Altman approach, only 4 data points now lie outside the upper/lower limits. As this equates to 2.7% of the data set, the Bland–Altman method (wrongly) accepts an hypothesis of agreement (Fig. 6). This is because the introduction of these new unexpected high/low values drives a significant increase in standard deviation and, thus, a change in the upper/lower limits of the model.

These new observations, combined with questions raised by other researchers [9, 10], lead us to believe that, whereas the Bland–Altman method is suitable for comparing homoscedastic methods, it does not provide additional useful information for methods exhibiting heteroscedastic behaviour and, in some cases, may lead to erroneous conclusions.

## Conclusion

When a total of 100 emulsion‐type sunscreen products (spanning SPF6 to 50+, comprising W/O, O/W and products with a mineral‐only sun filter system) were tested using the new double‐plate *in vitro* SPF test method, over 95% of paired *in vitro*: *in vivo* SPF values lay within the upper and lower limits of the ISO acceptance criteria funnel, with no bias.

This new *in vitro* SPF test method, therefore, meets the minimum requirements for an acceptable alternative SPF test method (to the current *in vivo* reference method, ISO24444:2010) for emulsion‐type sunscreen products (which make up the majority of marketed sunscreen products).
